# Effects of dry needling on spasticity, cortical excitability, and range of motion in a patient with multiple sclerosis: a case report

**DOI:** 10.1186/s13256-024-04452-z

**Published:** 2024-03-24

**Authors:** Haniyeh Choobsaz, Nastaran Ghotbi, Noureddin Nakhostin Ansari

**Affiliations:** 1 School of Rehabilitation, Department of Physiotherapy, Tehran University of Medical Sciences, Tehran, Iran; 2 Research Center for War-Affected People, Tehran University of Medical Sciences, Tehran, Iran

**Keywords:** Dry needling, Spasticity, Cortical excitability, Multiple sclerosis, Transcranial magnetic stimulation, Case report

## Abstract

**Background:**

Dry needling is an intervention used by physiotherapists to manage muscle spasticity. We report the effects of three sessions of dry needling on ankle plantar flexor muscle spasticity and cortical excitability in a patient with multiple sclerosis.

**Case presentation:**

The patient was a 40-year-old Iranian woman with an 11-year history of multiple sclerosis. The study outcomes were measured by the modified modified Ashworth scale, transcranial magnetic stimulation parameters, and active and passive ankle range of motion. They were assessed before (T0), after three sessions of dry needling (T1), and at 2-week follow-up (T2). Our result showed: the modified modified Ashworth scale was improved at T2 from, 2 to 1. The resting motor threshold decreased from 63 to 61 and 57 at T1 and T2, respectively. The single test motor evokes potential increased from 76.2 to 78.3. The short intracortical inhibition increased from 23.6 to 35.4 at T2. The intracortical facilitation increased from 52 to 76 at T2. The ankle active and passive dorsiflexion ROM increased ~ 10° and ~ 6° at T2, respectively.

**Conclusion:**

This case study presented a patient with multiple sclerosis who underwent dry needling of ankle plantar flexors with severe spasticity, and highlighted the successful use of dry needling in the management of spasticity, ankle dorsiflexion, and cortical excitability. Further rigorous investigations are warranted, employing randomized controlled trials with a sufficient sample of patients with multiple sclerosis.

*Trial registration* IRCT20230206057343N1, registered 9 February 2023, https://en.irct.ir/trial/68454

## Introduction

Spasticity is one of the most common symptoms in patients with multiple sclerosis (MS) [1]. It has been reported that approximately 97% of patients with MS have lower extremity spasticity, especially in the hip flexors and adductors, as well as in the knee flexors and ankle plantar flexors [2, 3]. Among the muscles of the lower extremities, several studies have shown that the triceps surae has an important function in balance and gait [4, 5]. Untreated spastic calf muscles can result in functional limitation with pain, limited joint mobility, and gait disturbance [6].

Dry needling (DN) was primarily used for musculoskeletal problems [7]. Today, it is also used to improve spasticity and the range of motion (ROM) in neurological conditions such as stroke, spinal cord injury, and MS [8–10]. The mechanisms by which DN can reduce spasticity is not completely known [11]. One possible is that DN regulates neuronal activity in the levels of spinal cord [12] or supraspinal centers [13, 14]. Recently, studies have shown that DN influences brain activity using functional magnetic resonance imaging (fMRI) [15, 16]. However, the effect of DN on cortical excitability is unclear. Cortical excitability reflects the responsiveness and response selectivity of cortical neurons to stimuli, reflects the reactivity and response specificity of neurons, and is therefore a fundamental aspect of human brain function [17]. Cortical excitability can be measured by transcranial magnetic stimulation (TMS) in several studies [18, 19]. TMS is a non-invasive technique to assess cortical changes and neuromodulation, especially in neurological diseases [20].

This case study uses TMS to evaluate the effects of three sessions of DN on cortical excitability as well as spasticity, and ankle range of motion (ROM) in a patient with relapsing–remitting MS.

## Case presentation

The patient was an Iranian 40-year-old woman who had a history of 11 years of relapsing–remitting MS and an expanded disability status scale (EDSS) of 2, and was able to walk independently. She had not experienced a relapse during the past 6 months. She complained of spasticity and functional impairment in her right lower extremity despite receiving physiotherapy, exercise therapy, and medication to alleviate spasticity. She had no history of comorbidities such as cardiovascular disease, diabetes, psychological problems, or familial diseases, and no contraindications for DN [21]. Her latest MRI report showed multiple plaques in cervical spinal cord and brain. The study was approved by the research ethics committee of Tehran University of Medical Sciences. The patient has given written and informed consent for the publication of this report and any accompanying images.

### Spasticity

Right leg plantar flexor spasticity was assessed by the Persian version of modified modified Ashworth scale (MMAS), a valid and reliable scale that rates the intensity of spasticity on a scale of 0–4 [22, 23]. With patient in a supine position and the legs extended, the physiotherapist passively moved the ankle from maximal plantarflexion to maximal dorsiflexion during 1 second counting one thousand and one and scored the resistance to passive stretch. (The patient has spasticity bilaterally in lower limbs, but right-side spasticity is more than the left).

### Passive and active ROM

Passive and active ROM of ankle dorsiflexion was measured using an ankle biplane goniometer (A Bissell Health Care, model 7524, USA) in the supine position, with the knee extended. Maximum passive ROM (PROM) was assessed by physiotherapist while passively moving the ankle to maximum dorsiflexion. Active ROM (AROM) was measured while the patient actively performed the dorsi flexion [24].

### Cortical excitability

**Fig. 1:**
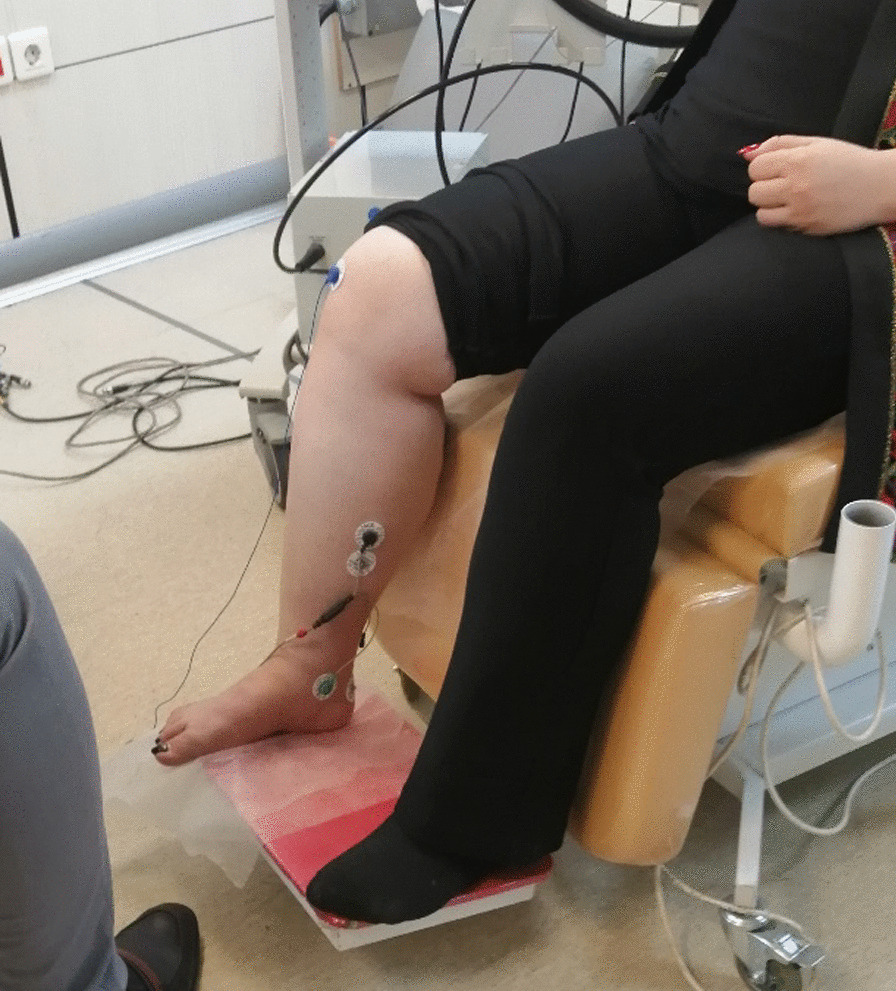
Electromyography electrodes for soleus muscle motor evoked potential record

Cortical excitability was assessed using motor-evoked potential (MEP), short interval intracortical inhibition (SICI), and intracortical facilitation (ICF). MEP represents the global excitability of the cortex, spinal, and corticospinal pathways [25]. SICI and ICF are well-known paired-pulse TMS that are used for investigating intracortical circuits in the motor cortex [26]. The resting motor threshold (RMT), expressed as a percentage of maximal stimulus output (%MSO), was measured using MagPro (MAG venture TMS, Denmark). The stimulation target location was fine-tuned for the patient to stimulate the right plantar muscle hotspot by an 8-shaped coil (coil head dimensions: 170 × 113 × 17.34 mm) defined as the optimal location for MEP in the contralateral spastic plantar flexor at the lowest stimulation intensity. MEP was recorded from soleus muscle electromyography (EMG) recordings (Seniam.org). The active and reference electrodes (20 mm apart) were placed on the main soleus muscle bulk near the motor point of the soleus muscle, located between the medial condyle of the femur and the medial malleolus. The electrodes were positioned at the ankle (around the medial malleolus (Fig. 1). The RMT was defined as the minimum TMS intensity required to produce detectable MEP amplitudes above 50 mv from background EMG in at least five out of ten trials. SICI refers to the phenomenon in which a subthreshold conditioning stimulus (CS) suppresses MEPs evoked by a subsequent suprathreshold test stimulus (TS) with a 3-ms inter-stimulus interval (ISI). ICF is a phenomenon of increased cortical excitability induced by conditioning stimuli and was assessed by test stimuli in a conditioning test paradigm. It was generated when a subthreshold CS occurs and stimulates MEPs evoked by a suprathreshold TS with an ISI of 13 ms (msec).

### Intervention

**Fig. 2:**
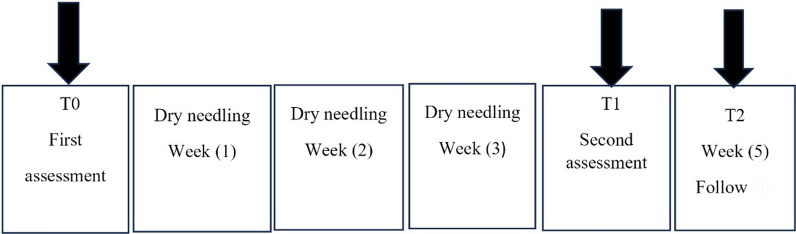
Timeline of interventions and assessments

**Fig. 3:**
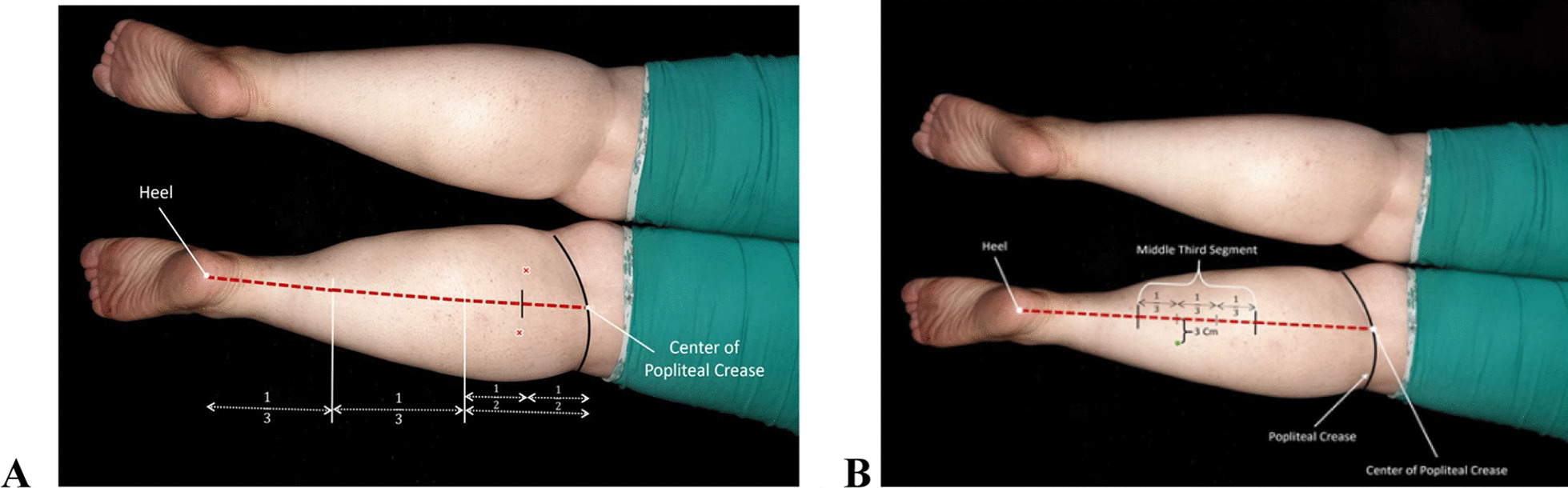
Points for dry needling of the gastrocnemius (**A**) and soleus (**B**)

We used a 0.3 mm × 50 mm (Korea) disposable stainless steel needle. A fast-in-fast-out technique was followed, and heads of gastrocnemius and soleus muscles were needled each for 1 minute (total 3 minutes). One DN session per week for 3 weeks was performed, and follow-up was carried out 2 weeks later (Fig. 2). For the gastrocnemius and soleus muscle, a line from the popliteal crease to the heel was drawn. This line was divided into three equal parts, and for the medial head of gastrocnemius about 2 cm medial from the middle of the proximal segment and about 2 cm lateral for the medial and lateral heads of gastrocnemius were needled [24]. For the soleus muscle, the middle segment of the line was also divided into three parts, and the needle was inserted about 2–3 cm lateral in the lower third part of the middle segment [27] (Fig. 3).

## Results

The spasticity of ankle plantar flexors decreased from 2 to 1 at T1 and remained unchanged at T2. Active dorsiflexion ROM increased after DN from 10° to 20° and passive dorsiflexion increased from 12° to 18°. The RMT decreased from 63 to 61 at T1 and 57 at T2. The changes in SICI were from 23.6 at T0 to 67.9 at T1 and 35.4 at T2. The ICF increased from 52 at T0 to 137 at T1 and 76 at T2. Table 1 presents the results before and after the intervention.

**Table 1:** Changes in measured parameters

Variables	Pre (T0)	Post-DN (T1)	Follow-up (T2)
MMAS	2	1	1
Passive DF ROM, degree	12	18	18
Active DF ROM, degree	10	20	20
RMT (%MSO)	63	61	57
MEP/mv	76.2	78.3	77.1
SICI 80%RMT/mv	23.6	67.9	35.4
ICF 120% RMT/mv	52	137	76

DN, dry needling; MMAS, modified modified Ashworth scale; DF, dorsiflexion; MSO, maximum stimulator output; RMT, resting motor threshold; SICI, short-interval intracortical inhibition; MEP, motor evoke potential; ICF, intracortical facilitation

## Discussion

In this case study involving a patient with MS, after three DN sessions targeting ankle plantar flexors, clinical and TMS measures improved. There was a decrease in RMT, accompanied by an increase in MEP amplitude. Changes in ICF indicated an increase in intracortical facilitation. Notably, the heightened cortical excitability persisted for 2 weeks following DN. These findings may be explained by the dual impact of DN, which excites the motor cortex through both peripheral sensory inputs and intracortical mechanisms [28, 29]. The DN, offering somatosensory conditioning stimulus, can generate excitability in the motor cortex through direct impact or cutaneous input from the spastic plantar flexor muscles, manifesting at short latency [28–30]. There is a possibility that somatosensory input from DN manipulation of spastic tissue contributes to an elevation in glutamate receptor concentration within the motor cortex, consequently augmenting ICF [31]. These results may prompt the hypothesis that heightened cortical excitability might play a role in reducing spasticity as reflected in significant improvement of spasticity in this patient with MS [32]. Our results showed the increase in ICF and MEP, and the decrease in RMT that indicated the increases in cortical excitability. Furthermore, SICI increased during the three sessions. The ICF/SICI ratio, as a parameter of excitability, also did not change noticeably. To justify these results, the following explanations should be noted. (1) The lower limb motor area in brain stimulation is very tiny in comparison with the upper extremity [33], so determining SICI (as an inhibition parameter with small amounts) is more difficult than usual studies. (2) Stimulation intensity and frequency can influence the ICF/SICI ratio parameter [34]. (3) Patient fatigue, as the most frequent problem in patients with MS, can influence increased SICI levels [35]. In the spasticity results, the reduction in spasticity observed in this MS case, in line with previous reports, validates the positive impact of DN on spasticity [9, 36, 37]. Improvements in ankle active and passive flexion ROM may be attributed to improvements in both spasticity and cortical excitability.

## Conclusion

This case study presented a patient with MS who underwent DN of ankle plantar flexors with severe spasticity, and highlighted the successful use of DN in the management of spasticity, ankle dorsiflexion, and cortical excitability. Further rigorous investigations are warranted, employing randomized controlled trials with a sufficient sample of patients with MS.

## Data Availability

All data generated or analyzed during this study are included.

## Abbreviations

MS: Multiple sclerosis
DN: Dry needling
ROM: Range of motion
SICI: Short interval intracortical inhibition
ICF: Intracortical facilitation
RMT: Rest motor threshold
